# International Statistical Classification of Diseases and data gaps in traditional medicine

**DOI:** 10.2471/BLT.25.293540

**Published:** 2025-10-14

**Authors:** Ye-Seul Lee, Yangmu Huang, Jon Wardle, Claudia M Witt, Myeong Soo Lee

**Affiliations:** a Jaseng Spine and Joint Research Institute, Jaseng Medical Foundation, Seoul, Republic of Korea.; b School of Public Health, Peking University, Beijing, China.; c National Centre for Naturopathic Medicine, Southern Cross University, Lismore, Australia.; d Chair for Complementary and Integrative Medicine, University of Zurich, Zurich, Switzerland.; e KM Science Research Division, Korea Institute of Oriental Medicine, 1672 Yuseong-daero, Yuseong-gu, Daejeon 34054, Republic of Korea.

Traditional, complementary and integrative medicine, as defined in the World Health Organization’s (WHO) new *Global traditional medicine strategy 2025–2034*,^1^ is widely used globally, yet remains weakly represented in routine data collection.^2^ In the absence of standardized data fields for traditional medicine diagnoses and encounters, policy-makers and researchers lack reliable insights into real-world use, costs, outcomes and harms, therefore hampering integration, regulation and evaluation within broader health systems.^3^ The *11th Revision of the international statistical classification of diseases and related health problems *(ICD-11) addresses part of this gap via the supplementary chapter for traditional medicine conditions (Chapter 26), which enables optional dual coding of traditional medicine disorders and patterns alongside conventional diagnoses.^4^^,^^5^

By providing an international code set for traditional medicine concepts, Chapter 26 is the ICD’s first formal mechanism for translating distinct diagnostic frameworks into a common statistical language, with the potential to support morbidity statistics and unlock further research towards evidence-informed strategies.^6^

In this article, we summarize the scope and evolution of Chapter 26; diagnose current data, evidence and policy gaps; highlight capabilities; identify limitations; and suggest a research–policy agenda.

## Scope and evolution

Although a clear majority of WHO Member States that provided information for the *WHO Global report on traditional and complementary medicine 2019* acknowledged population use of traditional medicine,^3^ most national databases have historically not coded these encounters, leaving service volume, safety signals and outcomes unmeasured. Many traditional medicine modalities are individualized and complex; therefore, randomized controlled trials alone do not provide the full picture. Recognizing this limitation, the 2023 WHO Traditional Medicine Summit in Gujarat, India,^5^ urged Member States to adopt real-world data approaches for evaluating traditional medicine. WHO highlighted the need for comparable coding frameworks;^7^ its main tool for this purpose is ICD-11 Chapter 26.

Chapter 26 builds on earlier standardization efforts, such as the establishment of standard nomenclature for acupoints by WHO and the International Classification of Traditional Medicine project, which harmonized disorders and patterns into a web-based classification system. In 2019, the World Health Assembly adopted ICD-11 including Chapter 26,^4^ which is a supplementary, morbidity-only chapter, enabling dual coding of traditional medicine concepts alongside conventional diagnoses. ICD-11 took effect on 1 January 2022 with Module I (East-Asian systems).^4^ Following the 2023 Summit’s call for standardized capture and real-world data evaluation, Module II (Ayurveda, Siddha, Unani) was added in 2025, extending the scope beyond East-Asian traditions.^8^ A joint task force of clinicians, researchers and WHO classification specialists reconciled national terminologies into a clinically meaningful, linguistically interoperable core set.

## Current gaps

Data on traditional medicine is structurally under-captured, and terminology is heterogeneous. Electronic health records and insurance claims have lacked structured fields for traditional medicine diagnoses and interventions. Consultations were often unrecorded or embedded in free text, precluding aggregation and comparative analysis.^9^ Cross-national differences in practice-specific diagnostic terminologies further complicated mapping; even systems sharing East-Asian traditional medicine concepts have used non-interchangeable coding schemes, making one-to-one mappings to biomedical taxonomies error-prone, as they lack careful semantics and versioning. Methodological work on ICD-11 reveals that Chapter 26 was introduced precisely to mitigate cross-country semantic drift and enable dual coding.^7^

In the absence of structured routine data, research is constrained and, therefore, traditional medicine research has been heavily relying on population surveys that offer utilization snapshots but rarely include harmonized cost or outcome measures, limiting cross-country comparability. Safety assessment is undermined when adverse events related to medicinal plants or procedures bypass standard pharmacovigilance channels. The 2023 Summit proceedings explicitly called for stronger real-world data infrastructure and better linkages to safety reporting to close these gaps.^5^

In the absence of data, policy formulation and future practice are hampered, as traditional medicine usage remains invisible. Health insurance coverage for traditional medicine treatments and therapies drives coding: only what the government recognizes and reimburses is recorded, leading to further invisibility of traditional medicine usage and underreporting. In Member States where traditional medicine is integrated and reimbursed, medical data are more visible;^9^^,^^10^ where coverage is limited, underreporting is systematic.^11^^,^^12^ Although Chapter 26 provides a data tool to align coverage when activated, its supplementary and optional status means that ICD-11 adoption does not guarantee its use. Member States that choose to defer Chapter 26 coding will exhibit adoption-utilization asymmetry where routine data stay silent while private-sector traditional medicine use persists.

## Capabilities 

Chapter 26 addresses the above gaps at the classification layer, since it distinguishes traditional medicine disorders (disease entities) from traditional medicine patterns (functional imbalances identified through traditional diagnostics).^4^ Dual coding permits a biomedical diagnosis to be paired with its interpretation in traditional medicine concepts.^7^ Chapter 26 is implemented on the same digital architecture as the rest of ICD-11. Therefore, clustered codes can be represented in the same Fast Healthcare Interoperability Resources (which is an international standard for electronically exchanging health-care data) along with conventional codes, enabling integrated analytics. When paired with ICD-11 extension codes and International Classification of Health Interventions procedure codes, this infrastructure code can support pharmacovigilance (such as tracking medicinal plant–drug interactions or procedure-related harms) and longitudinal utilization analyses. The ICD-11 implementation guide outlines how health ministries can incorporate Chapter 26 into surveillance, reimbursement and quality-monitoring workflows.^6^ By bridging diverse knowledge systems into a shared statistical language, Chapter 26 allows traditional medicine to be countable and comparable in routine data.

## Limitations

Notwithstanding these capabilities, several factors may impede translation into comparable statistics or better care. First, optionality creates adoption-utilization asymmetry: some systems, especially in some high-income countries, may adopt ICD-11 for core chapters yet ignore Chapter 26, yielding regionally biased statistics.^13^ Second, interoperability gaps remain: practical linkages to the International Classification of Health Interventions (procedures), SNOMED CT (clinical terminology) and Fast Healthcare Interoperability (exchange profiles) require curated mappings and clear documentation of non-equivalences. Without these, data exchange and decision support will be limited. Third, validity is not guaranteed: even within the same system, different practitioners may not agree when assigning traditional medicine patterns; and across systems, the underlying concepts may not align. Validity must be demonstrated before multinational comparisons are relied upon. Finally, policy and reimbursement dependencies mean that coverage decisions will shape what is coded, and therefore private-sector activity may remain invisible.

## Future directions 

Realizing the potential of Chapter 26 requires both adopting and using the codes in practice, which in turn involves building analytics and governance by creating policy frameworks, regulatory oversight and institutional accountability to ensure that codes are applied and monitored. Countries should establish utilization metrics that separately measure ICD-11 adoption and Chapter-26 use (such as dual-coding rate among eligible encounters, facility and department uptake, volumes by disorder or pattern, geographic distribution). National statistics should make such gaps visible and guide remediation. Interoperability can be strengthened through several measures. First, by standardizing links to the International Classification of Health Interventions for procedures, such as details of acupuncture sessions. Second, by publishing Fast Healthcare Interoperability implementation guides that include ICD-11 clusters, including those from Chapter 26, alongside encounter and medication resources. Third, where relevant, by developing SNOMED CT reference sets and mapping rules for high-value concepts, with explicit notes on non-equivalence to support decision-making and cross-system analytics.

The validity and reliability of Chapter 26 require dedicated study. Within-system reliability should be tested through interrater and test–retest assessments among traditional medicine clinicians, while between-system validity should examine convergent and predictive relationships between traditional medicine patterns across multiple Member States. Results will allow an evaluation of cross-walk performance across languages and locales so that multinational statistics are interpretable and robust.

Policy and reimbursement audits are essential to understand real-world use. Health insurance systems should conduct periodic reviews of payer policies, fee schedules and claim streams to assess whether Chapter 26 is referenced in coverage rules, how often dual-coded encounters are billed, and whether payment policies alter utilization, access or equity; comparisons across public and private payers can reveal unintended incentives. Finally, linkage to outcomes and harms must be built in from the outset. Pre-registered real-world studies should connect traditional medicine codes to clinical outcomes, utilization and costs, and safety signals by integrating electronic health records, claims and relevant registries.

To enable these steps, health ministries and WHO collaborating centres should pilot Chapter 26 in representative hospitals, primary-care clinics and dedicated traditional medicine facilities to assess data quality, workflow impact, coder agreement and interoperability before scaling up. Capacity-building is essential: joint curricula for coders, informaticians, traditional medicine practitioners and claims officers should accompany system integration. Clear manuals, high-quality translations and application across modules should ensure that data use enhances rather than marginalizes traditional medicine services.

Chapter 26 closes a long-standing information gap by developing a common, interoperable language for traditional medicine disorders and patterns. Incorporating Chapter 26 into national data standards will allow traditional medicine providers to encode medical encounters and researchers to analyse the resulting data sets. Practices that were once difficult to track can progress to measurable evidence, guiding the system towards data-driven policy and care. This shift will enable more precise regulation, financing and integration of diverse systems, advancing WHO’s vision of people-centred, evidence-informed health care. Achieving better health for all thus requires better data for all, traditional medicine included.
